# Temozolomide for the treatment of metastatic melanoma

**DOI:** 10.3747/co.2007.98

**Published:** 2007-02

**Authors:** I. Quirbt, S. Verma, T. Petrella, K. Bak, M. Charette, the members of the Melanoma Disease Site Group of Cancer Care Ontario’s Program in Evidence-Based Care

**Affiliations:** * Cancer Care Ontario Program in Evidence-Based Care, McMaster University, Hamilton, Ontario; † Princess Margaret Hospital, Toronto, Ontario.; ‡ The Ottawa Hospital, Ottawa, Ontario.; § Sunnybrook Regional Cancer Center, Toronto, Ontario; || Please see the Web site of Cancer Care Ontario’s Program in Evidence-Based Care (www.cancercare.on.ca/access_PEBC.htm) for a complete list of current Melanoma Disease Site Group members.

**Keywords:** Melanoma, temozolomide, temodal, guideline report

## Abstract

**Questions:**

What is the role of single-agent temozolomide in the treatment of patients with metastatic melanoma?

In comparison with single-agent temozolomide, does the addition of interferon-α to temozolomide improve disease-free survival, overall survival, or response rates?

In comparison with single-agent temozolomide, does the addition of thalidomide to temozolomide improve disease-free survival, overall survival, or response rates?

**Perspectives:**

Because of its oral route of administration and its ability to cross the blood–brain barrier, temozolomide is a potentially attractive chemotherapy agent for adult patients with unresectable metastatic malignant melanoma. To provide treatment recommendations for this new agent, the Melanoma Disease Site Group (dsg) of Cancer Care Ontario’s Program in Evidence-Based Care (pebc) decided to review the available literature on single-agent temozolomide and on temozolomide in combination with interferon-α or thalidomide.

**Outcomes:**

Outcomes of interest included response rates, disease-free survival, overall survival, quality of life, and adverse effects.

**Methodology:**

Evidence was selected and reviewed by two members of the Melanoma dsg and by methodologists. The present practice guideline report was reviewed and approved by the Melanoma dsg, which comprises medical and radiation oncologists, surgeons, and dermatologists. External review was obtained through a mailed survey of Ontario practitioners, the results of which were reflected in revisions to the practice guideline. Final approval of the guideline report was obtained from the Report Approval Panel of the pbec.

**Practice Guideline:**

These recommendations apply to adult patients with unresectable metastatic malignant melanoma.

It is reasonable to use temozolomide at a dose of 200 mg/m^2^ orally for 5 days every 4 weeks as initial systemic treatment for patients with unresectable metastatic malignant melanoma.

The addition of moderate-dose interferon-α 2b has produced a significantly higher response rate than has single-agent temozolomide in a large randomized phase iii study. However, overall survival was not altered, and grades 3 and 4 hematologic toxicities were higher with the combined treatment. At the present time, the addition of interferon-α to temozolomide is not recommended.

One randomized phase ii study and six other phase ii studies showed encouraging response rates when thalidomide was combined with temozolomide. However, the doses and schedules of temozolomide in those studies differed from the conventionally prescribed doses and schedules. It is not clear whether the improved response rates were attributable to the small number of patients in the studies, the different temozolomide doses and schedules, or the addition of thalidomide. Further phase iii studies are required to confirm whether a benefit is associated with the combination of temozolomide and thalidomide. Therefore, at this time, it is not recommended that thalidomide be combined with temozolomide.

**Qualifying Statements:**

Dacarbazine is the only chemotherapy drug currently approved for the treatment of metastatic malignant melanoma. In large randomized trials, response rates with dacarbazine ranged from 6% to 15%. Almost all responses were partial, with a median response duration of only 7–8 months. Given these disappointing overall results, the consensus among most physicians who are treating patients with metastatic malignant melanoma is that recommending more convenient treatment or experimental treatment to these patients is appropriate.

Because of oral dosing, temozolomide is a reasonable choice, particularly for patients who would have difficulty traveling to cancer centres for intravenous chemotherapy.

Temozolomide has demonstrated efficacy equal to that of dacarbazine in a randomized phase iii trial. However, unlike dacarbazine, temozolomide is a convenient oral treatment that penetrates the blood–brain barrier and that has shown activity against brain metastases. Although surgery is the preferred treatment modality for patients with solitary brain metastases from melanoma, temozolomide is the preferred chemotherapy for patients with brain metastases who require systemic treatment.

## 1. QUESTIONS

What is the role of single-agent temozolomide in the treatment of patients with metastatic melanoma?

In comparison with single-agent temozolomide, does the addition of interferon-α to temozolomide improve disease-free survival, overall survival, or response rates?

In comparison with single-agent temozolomide, does the addition of thalidomide to temozolomide improve disease-free survival, overall survival, or response rates?

Outcomes of interest included response rates, disease-free survival, overall survival, quality of life, and adverse effects.

## 2. CHOICE OF TOPIC AND RATIONALE

It has been estimated that, during 2005, 4400 new cases of melanoma will have been diagnosed in Canada and that, of 880 estimated deaths from the disease, 420 will have occurred in Ontario 1. Primary surgical treatment cures most patients diagnosed with early-stage malignant melanoma. However, people with deeply invasive melanoma have a high probability of developing distant metastases. Adjuvant therapy has been found to be partially effective, but the currently available systemic treatments have yielded disappointing results.

Recently, temozolomide has emerged as a promising novel chemotherapy agent in malignant melanoma. Temozolomide acts through the same mechanism as dacarbazine; however, unlike dacarbazine, temozolomide has excellent oral bioavailability and possesses the ability to cross the blood–brain barrier. These added benefits suggest that temozolomide may play a role in treating patients with metastases to the brain, a frequent metastatic site in melanoma.

To provide treatment recommendations for this new agent, the Melanoma Disease Site Group (dsg) decided to review the available literature on single-agent temozolomide and on temozolomide in combination with interferon-α or thalidomide.

## 3. METHODS

### 3.1 Guideline Development

This practice guideline report was developed by Cancer Care Ontario’s Program in Evidence-Based Care (pebc) using the methods of the practice guidelines development cycle 2. Evidence was selected and reviewed by two members of the Melanoma dsg and by methodologists. Members of the Melanoma dsg disclosed potential conflict of interest information.

The practice guideline is a convenient and up-to-date source of the best available evidence on single-agent temozolomide or temozolomide in combination with interferon-α or thalidomide in the treatment of metastatic melanoma. The present report was developed through systematic reviews, evidence synthesis, and input from practitioners in Ontario. It is a companion piece to a systematic review which is currently under consideration for publication elsewhere. Both documents are intended to promote evidence-based practice in Ontario, Canada. The pebc is editorially independent of Cancer Care Ontario and the Ontario Ministry of Health and Long-Term Care.

### 3.2 Literature Search Strategy

The medline [1966 to September (week 3) 2005], embase (1980 to week 40, 2005), and the Cochrane Library (Issue 3, 2005) were systematically searched using a combination of the following terms: “melanoma” [Medical Subject Heading, *Excerpta Medica* Tree (emtree) term, and text word], “temozolomide” (emtree term and text word), “temodal” (text word), and “temodar” (text word). Those terms were then combined with the search terms for the following publication types and study designs: practice guidelines, systematic reviews, meta-analyses, randomized controlled trials, and controlled trials.

In addition, the proceedings of the 1997–2005 annual meetings of the American Society of Clinical Oncology were searched for reports of newly completed trials. Relevant articles and abstracts were selected and reviewed by two reviewers, and the reference lists from those sources were searched for additional trials, as were the reference lists from relevant review articles.

## 4. RESULTS

### 4.1 Literature Search

The literature search identified two randomized phase iii trials and three randomized phase ii trials of single-agent temozolomide compared with either single-agent intravenous dacarbazine or combinations of temozolomide with cisplatin, interferon-α 2b (ifn), or thalidomide. Another nine phase ii and phase i trials investigating single-agent temozolomide, six trials investigating temozolomide plus interferon-α, and six trials investigating temozolomide plus thalidomide were reviewed. Sixteen of the trials were published as full reports; ten were available in abstract form only.

### 4.2 Outcomes

#### 4.2.1 Randomized Phase III Trials

Evidence from the two randomized trials 3,4 shows that it is reasonable to use temozolomide as initial treatment for patients with unresectable metastatic malignant melanoma. The first trial 3 compared single-agent temozolomide with single-agent intravenous dacarbazine in 305 patients. The second trial 4 compared single-agent temozolomide with temozolomide combined with ifn in 294 patients.

Results from the first trial, by Middleton *et al.,* demonstrated that progression-free survival was significantly prolonged in patients treated with temozolomide, with a reported hazard ratio (hr) of 1.37 [95% confidence interval (ci): 1.07 to 1.75; *p* = 0.012]. Median survival for patients treated with temozolomide was 7.7 months versus 6.4 months for those treated with dacarbazine. However, the difference between the two treatment groups was not statistically significant. Of the patients in the temozolomide arm, 21 (14%) showed an objective response to treatment; of patients in the dacarbazine arm, 18 (12%) showed an objective response. Grades 3 and 4 hematologic toxicities were similar in the two treatment arms.

Kaufmann *et al.* 4 reported a significantly higher response rate for temozolomide combined with ifn as compared with single-agent temozolomide (24% vs. 13%; *p* < 0.036). In the combination arm, 11 patients (8%) achieved a complete response, and 22 patients (16%) showed a partial response, as compared with 3 patients (2%) and 15 patients (11%) respectively in the temozolomide group. The median overall survival was reported to be 8.4 months in the temozolomide group (95% ci: 7.07 to 9.72) and 9.7 months in the temozolomide plus ifn group (95% ci: 8.26 to 11.18); no statistical significance was detected.

#### 4.2.2 Randomized Phase II Trials

The systematic review identified three randomized phase ii trials 5–7. In the first trial 5, 127 patients were randomized to receive temozolomide alone or in combination with cisplatin. The second trial 6 randomized 181 patients to receive temozolomide alone or temozolomide combined with either ifn or thalidomide. The third trial 7 randomized 47 patients to a treatment combination of temozolomide and ifn, the latter being administered in two different dosages. None of trials detected statistically significant differences in median overall survival, time to disease progression, or objective response.

Interestingly, the trial 5 by Bafaloukos and colleagues detected some evidence of antitumour activity in the central nervous system, including 3 partial responses in brain metastases (1 in the temozolomide arm and 2 in the combination arm). Only 16% of patients receiving temozolomide alone and 18% of patients receiving temozolomide with cisplatin developed central nervous system metastases (median follow-up of 39.9 months and 37 months, respectively), further suggesting that treatment with temozolomide may reduce the occurrence of brain metastases.

#### 4.2.3 Single-Arm Phase II and I Trials

Phase ii and i trials of single-agent temozolomide have demonstrated response rates that range from 0% to 29%, with complete responses observed in 0%–17% of patients. Phase ii and i trials investigating temozolomide in combination with either ifn or thalidomide have reported response rates ranging from 13% to 23% and from 8% to 42% respectively. Although these response rates are encouraging, further evidence from randomized trials is required.

## 5. DSG CONSENSUS PROCESS

The practice guideline recommendations were drafted by one member of the Melanoma dsg and circulated to the entire dsg in February 2004 for review and consensus. The members of the Melanoma dsg unanimously agreed with the draft recommendations. Before submitting the recommendations to the Report Approval Panel, the dsg members discussed adding a recommendation regarding the use of temozolomide for patients with brain metastases who require systemic treatment. However, after some debate, the members decided to address that issue in a qualifying statement.

## 6. PRACTITIONER FEEDBACK

### 6.1 Methods

Following discussion and consensus, the Melanoma dsg circulated the clinical practice guideline and systematic review to clinicians in Ontario for review. Feedback was obtained through a mailed survey of 13 practitioners in Ontario (all medical oncologists). The survey consisted of items evaluating the methods, results, and interpretive summary used to inform the draft recommendations and asking whether the draft recommendations should be approved as a practice guideline. Written comments were invited. The survey was mailed on April 23, 2004. Follow-up reminders were sent at 2 weeks (post card) and 4 weeks (complete package mailed again). The Melanoma dsg reviewed the results of the survey.

### 6.2 Results

Of the 13 practitioners surveyed, 8 (62%) responded. Responses include returned completed surveys and telephone, fax, and e-mail responses. Of the practitioners who responded, 6 indicated that the report was relevant to their clinical practice, and they completed the survey. All practitioners indicated that they agreed with the draft recommendations as stated, and most agreed that the document should be approved as a practice guideline. Table I summarizes key results of the practitioner feedback survey.

### 6.3 Summary of Written Comments

Four respondents (67%) provided written comments. The main points contained in the written comments were these:

- One practitioner commented that experience with the 6-week 50–75 mg/m^2^ daily regimen is adequate to include it as a reasonable treatment option.
- Another practitioner commented that temozolomide is a reasonable choice of therapy only insofar as dacarbazine is reasonable. Dacarbazine should be the “default” option, and temozolomide should be used only under unusual circumstances—for example, when patients live in remote areas or lack access to intravenous treatment—and even then, monitoring the patient’s counts is important.
- Finally, a practitioner commented that the roles of interferon and thalidomide should be revisited in a timely manner. Also, a need exists to address the funding of temozolomide in a timely manner so that it is accessible to patients.

### 6.4 Modifications/Actions

The Melanoma dsg discussed the comments resulting from practitioner feedback survey and responded as follows:

- Although experience with the 6-week 50–75 mg/m^2^ daily regimen is growing, the evidence is currently insufficient to endorse or adopt this treatment regimen in metastatic melanoma.
- Dacarbazine and temozolomide may both be considered reasonable therapeutic choices in patients. There is no reason to choose one over the other insofar as outcomes (overall survival, disease-free survival) are concerned. Patient preference and factors such as intravenous access, geographic location, and access to treatment facilities should be taken into consideration.
- As previously acknowledged in this document, the amount of high-quality evidence on the roles of ifn and thalidomide in combination with temozolomide is limited. To date, only one small rct has shown that the combination of temozolomide and ifn demonstrates a statistically significant difference in response rate. Perhaps the ongoing phase ii trial (Southwest Oncology Group S0508) comparing thalidomide and temozolomide in stage iv malignant melanoma will provide favourable results for this combination treatment. When larger confirmatory trials become available, the guideline will be updated and the recommendations revisited. The Melanoma dsg previously submitted this practice guideline report to the Drug Quality and Therapeutics Committee/Cancer Care Ontario (dqtc/cco) in 2005 for funding consideration.

## 7. REPORT APPROVAL PANEL

### 7.1 Results

After the practitioner feedback survey had been addressed, the practice guideline report was circulated to the pebc Report Approval Panel for further review. The Panel consists of two members, including an oncologist with expertise in clinical and methodology issues. The final report was reviewed and approved by the Panel in March 2006 on condition that the Melanoma dsg address these concerns:

- The justification for recommending temozolomide appears to be based on the fact that it meets the “benchmark” set by dacarbazine.
- Given the existing phase iii and phase ii studies, the inclusion of single-arm trials has the potential to bias discussion.

### 7.2 Modifications/Actions

In response, the Melanoma dsg

- acknowledged the view that dacarbazine is widely regarded as the standard of care—albeit one with extremely limited efficacy in this disease. Furthermore, the dsg members agree that temozolomide at least meets this standard and may be particularly applicable in the circumstances outlined in the report. Given that the “standard” leaves much to be desired, the dsg recommends that untreated patients be strongly considered for clinical trials. A statement on dacarbazine has been added to the discussion.
- The Melanoma dsg retained the single-arm trials because the limited evidence available made their inclusion appear necessary. The dsg members realize that the results of these studies must be interpreted with caution because of their methodologic limitations, and they plan to refrain from including them in future documents.

## 8. PRACTICE GUIDELINE

This practice guideline reflects the integration of the draft recommendations with feedback obtained during the external review process. It has been approved by the Melanoma dsg and the Report Approval Panel of the pbec.

### 8.1 Target Population

The recommendations in this practice guideline apply to adult patients with unresectable metastatic malignant melanoma.

### 8.2 Recommendations

Based on a systematic review of the available evidence, the Melanoma dsg concludes that it is reasonable to use temozolomide at a dose of 200 mg/m^2^ orally for 5 days every 4 weeks as initial systemic treatment for patients with unresectable metastatic malignant melanoma.

In a large randomized phase iii study, the addition of moderate-dose ifn has produced a significantly higher response rate than has single-agent temozolomide. However, overall survival was not altered and grades 3 and 4 hematologic toxicities were higher with the combined treatment. At the present time, the addition of interferon-α to temozolomide is not recommended.

One randomized phase ii study and six other phase ii studies showed encouraging response rates when thalidomide was combined with temozolomide. However, the doses and schedules of temozolomide in those studies differed from the conventionally prescribed doses and schedules. It is not clear whether the improved response rates were attributable to the small number of patients in the studies, the different temozolomide doses and schedules, or the addition of thalidomide. Further phase iii studies are required to confirm whether a benefit is associated with the combination of temozolomide and thalidomide. Therefore, at this time, it is not recommended that thalidomide be combined with temozolomide.

### 8.3 Qualifying Statements

Dacarbazine is the only chemotherapy drug currently approved for the treatment of patients with metastatic malignant melanoma. In large randomized trials, response rates with dacarbazine ranged from 6% to 15%. Almost all responses were partial, with a median response duration of only 7–8 months. Given these disappointing overall results, the consensus among most physicians who are treating patients with metastatic malignant melanoma is that recommending more convenient treatment or experimental treatment to these patients is appropriate.

Because of oral dosing, temozolomide is a reasonable choice, particularly for patients who would have difficulty travelling to cancer centres for intravenous chemotherapy.

Temozolomide has demonstrated efficacy equal to that of dacarbazine in a randomized phase iii trial. However, unlike dacarbazine, temozolomide is a convenient oral treatment that penetrates the blood–brain barrier and that has shown activity against brain metastases. Although surgery is the preferred treatment modality for patients with solitary brain metastases from melanoma, temozolomide is the preferred chemotherapy for patients with brain metastases who require systemic treatment.

## 9. PRACTICE GUIDELINE DATE

Completed March 2006. Practice guidelines developed by the pbec are reviewed and updated regularly. Please visit the pebc’s Web site (www.cancercare.on.ca/access_PEBC.htm) for the full guideline report and subsequent updates.

## Figures and Tables

**TABLE I:** Practitioner responses to eight items on the practitioner feedback survey

	*Respondents who [*n *(%)]*		
*Item*	*Strongly agree or agree*	*Neither agree nor disagree*	*Strongly disagree or disagree*
The rationale for developing a clinical practice guideline, as stated in the “Choice of Topic” section of the report, is clear.	6 (100)	0	0
There is a need for a clinical practice guideline on this topic.	6 (100)	0	0
The literature search is relevant and complete.	6 (100)	0	0
The results of the trials described in the report are interpreted according to my understanding of the data.	6 (100)	0	0
The draft recommendations in this report are clear.	6 (100)	0	0
I agree with the draft recommendations as stated.	6 (100)	0	0
This report should be approved as a practice guideline.	5 (83)	1 (17)	0

	*Very likely or likely*	*Unsure*	*Not at all likely or unlikely*

If this report were to become a practice guideline, how likely would you be to make use of it in your own practice?	4 (67)	0	2 (33)
